# Supplementation of SkQ1 Increases Mouse In Vitro Oocyte Maturation and Subsequent Embryonic Development by Reducing Oxidative Stress

**DOI:** 10.3390/ph17040455

**Published:** 2024-04-02

**Authors:** Zheng Li, Yiqian Zhang, Jinping Cao, Xupeng Xing, Yalin Liang, Yuxing Zhang, Xiaopeng Tang, Shengyi Lin, Zhenfang Wu, Zicong Li, Sixiu Huang

**Affiliations:** 1 National Engineering Research Center for Breeding Swine Industry, South China Agricultural University, Guangzhou 510642, China; lizheng2022@stu.scau.edu.cn (Z.L.); zyqhah-@stu.scau.edu.cn (Y.Z.); caojinping2021@stu.scau.edu.cn (J.C.); xupengxing@scau.edu.cn (X.X.); yalin_liang@stu.scau.edu.cn (Y.L.); zhangyuxing1998@163.com (Y.Z.); hawk@stu.scau.edu.cn (X.T.); linsy@stu.scau.edu.cn (S.L.); wzf@scau.edu.cn (Z.W.); 2 State Key Laboratory of Swine and Poultry Breeding Industry, South China Agricultural University, Guangzhou 510642, China; 3 National and Local Joint Engineering Research Center for Livestock and Poultry Breeding Industry, South China Agricultural University, Guangzhou 510642, China; 4 Department of Animal Genetics, Breeding and Reproduction, College of Animal Science, South China Agricultural University, Guangzhou 510642, China; 5 Guangdong Provincial Key Laboratory of Agro-Animal Genomics and Molecular Breeding, South China Agricultural University, Guangzhou 510642, China; 6 Gene Bank of Guangdong Local Livestock and Poultry, South China Agricultural University, Guangzhou 510642, China

**Keywords:** SkQ1, antioxidant, in vitro oocyte maturation, assisted reproduction

## Abstract

In vitro oocyte maturation (IVM) technology is important for assisted animal and human reproduction. However, the maturation rates and developmental potential of in vitro-matured oocytes are usually lower than those of in vivo-matured oocytes. Oxidative stress is a main factor that causes the lower maturation rates and quality of in vitro-matured oocytes. The purpose of this study was to investigate the effects of treatment with SkQ1, a mitochondria-targeted antioxidant, on mouse IVM and subsequent embryonic development. The results demonstrated that the supplementation of SkQ1 during IVM improves the maturation rates of mouse oocytes and the subsequent developmental competence of in vitro-fertilized embryos. The addition of SkQ1 to the IVM medium also decreased oxidative stress and apoptosis, and increased mitochondrial membrane potential in matured mouse oocytes. This study provides a new method through which to enhance the maturation rates and the quality of in vitro-matured mouse oocytes, thus promoting the application and development of assisted animal and human reproductive technology.

## 1. Introduction

In vitro oocyte maturation (IVM) technology is widely utilized in both assisted human reproduction and the animal husbandry industry. IVM offers fertility possibilities for patients with disorders such as ovarian hyperstimulation syndrome (OHSS) and ovarian cancers [1]. IVM also contributes to generating a substantial number of oocytes for various animal embryo biotechnologies, including cloning and transgenesis. However, in vitro-matured oocytes usually have lower maturation rates, poorer quality, and lower fertilization rates than in vivo-matured oocytes [2]. The low developmental potential of in vitro-matured oocytes significantly constrains the advancement of assisted human and animal reproduction [3].

Oxidative stress is one of the most significant factors that negatively affects the in vitro maturation of oocytes. Elevated oxygen levels, coupled with the absence of protective free radical scavengers and antioxidants, in the in vitro maturation environment, in contrast to in vivo conditions, contribute to a redox imbalance, inducing oxidative stress in in vitro-matured oocytes [4,5,6]. This stress usually impairs mitochondrial function [7], leading to increased levels of reactive oxygen species (ROS), increased expression of apoptotic factors, and decreased glutathione (GSH) levels, and triggering apoptosis [2,8]. Mitochondria generate ATP through electron transport chain (ETC)-coupled oxidative phosphorylation (OXPHOS), while concurrently producing ROS [8]. Approximately 90% of ROS in cells are generated from mitochondria, and excessive production of ROS can cause mitochondrial dysfunction and a decline in ATP, among other adverse effects [9,10]. Additionally, mitochondrial DNA (mtDNA), which are not protected by histones, are more susceptible than nuclear DNA to damage, due to excessive ROS production [4]. Continuous accumulation of mtDNA mutations leads to ETC dysfunction, generating more ROS and forming a vicious cycle [11]. Therefore, reducing oxidative stress is crucial for IVM. 

In recent years, considerable research efforts have been dedicated to eliminating oxidative stress during IVM. Studies indicate that melatonin [12] exhibits the capacity to enhance oocyte developmental competence through the scavenging of free radicals, activating diverse antioxidant mechanisms, and enhancing mitochondrial functionality [13]. Additionally, antioxidants such as vitamin E, vitamin C [14], and quercetin [15] have been shown to have beneficial effects on oocyte maturation, and subsequent embryonic development, in mice.

SkQ1 (plastoquinonyl-decyl-triphenylphosphonium), a conjugate of a lipophilic decyltriph-enylphosphonium cation with an antioxidant moiety of a plastoquinone, is a novel mitochondria-targeted antioxidant [16]. It can penetrate the cell membrane and enter the inner mitochondrial membrane aggregation, reducing damage to cells by preventing the overproduction of ROS [17]. Furthermore, SkQ1 can be rapidly reduced by mitochondrial respiratory chain complexes I and II, rendering it a reusable antioxidant [18]. However, SkQ1 has not been used in IVM so far.

The objective of this study was to investigate the effects of the addition of SkQ1 to mouse IVM medium on the oocyte maturation rate and subsequent embryonic developmental competence. The impacts of SkQ1 treatment on intracellular GSH and ROS levels, mitochondrial membrane potential, and the expression of apoptosis- and oxidative stress-related genes were also assessed in in vitro-mated oocytes.

## 2. Results

### 2.1. SkQ1 Improved the Maturation Rates and Developmental Potential of In Vitro-Matured Mouse Oocytes

**Figure 1:**
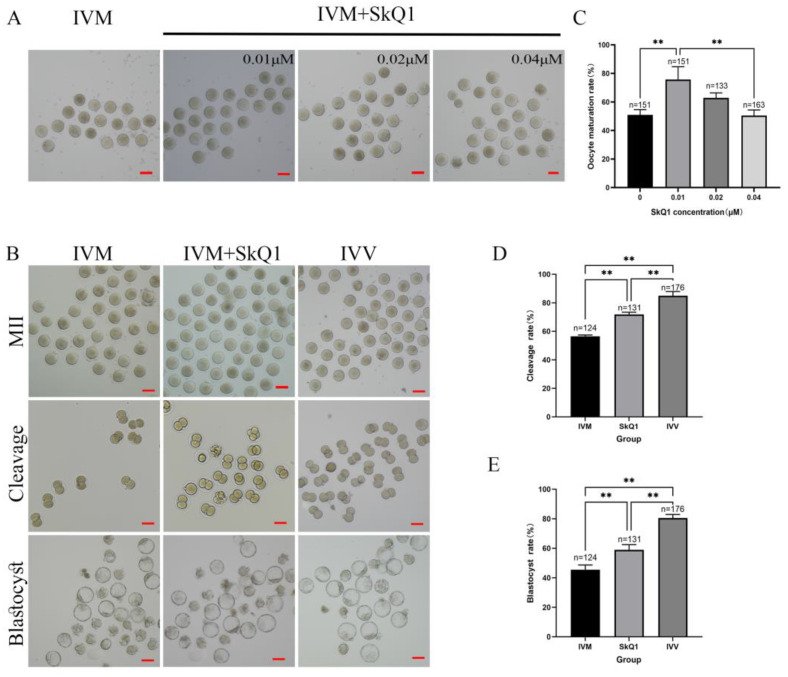
Effects of SkQ1 on mouse oocyte maturation and developmental potential. (**A**) Representative images of MII-stage oocytes after treatment with different concentrations of SkQ1. (**B**) Representative images of MII-stage oocytes, and cleavage-stage and blastocyst-stage IVF embryos. (**C**) Maturation rate of each group of oocytes. (**D**) Cleavage rate of each group of IVF embryos. (**E**) Blastocyst rate of each group of oocytes. “n” represents the number of analyzed oocytes or IVF embryos in each group. ** represents *p* < 0.01. Scale bar: 50 μm. IVM—in vitro-matured oocytes or IVF embryos generated from them; IVM + SkQ1—SkQ1-treated in vitro-matured oocytes or IVF embryos generated from them; IVV—in vivo-matured oocytes or IVF embryos generated from them.

Various doses of SkQ1 (0.01 μM, 0.02 μM, and 0.04 μM) were added to the IVM medium with mouse oocytes to study their effects on maturation rate. The results revealed a significant enhancement in oocyte maturation rate with the addition of 0.01 μM SkQ1 (Figure 1A,C). To further assess the impact of SkQ1 on oocyte developmental ability, in vitro fertilization (IVF) experiments were conducted. The results demonstrated that the group of IVF embryos produced from 0.01 μM SkQ1-treated in vitro-matured oocytes (IVM + SkQ1 group) exhibited a significantly higher cleavage rate and blastocyst rate than the control group (IVM group), although their developmental indexes were still lower than those of the in vivo-matured oocyte-derived IVF embryos (IVV group) (Figure 1B,D,E). These results suggest that the supplementation of 0.01 µM SkQ1 during the IVM process can effectively improve both the maturation and quality of mouse oocytes. Therefore, the concentration of 0.01 µM SkQ1 was used in the subsequent experiments.

### 2.2. SkQ1 Reduced Oxidative Stress Levels and Enhanced Antioxidant Ability in In Vitro-Matured Mouse Oocytes

**Figure 2:**
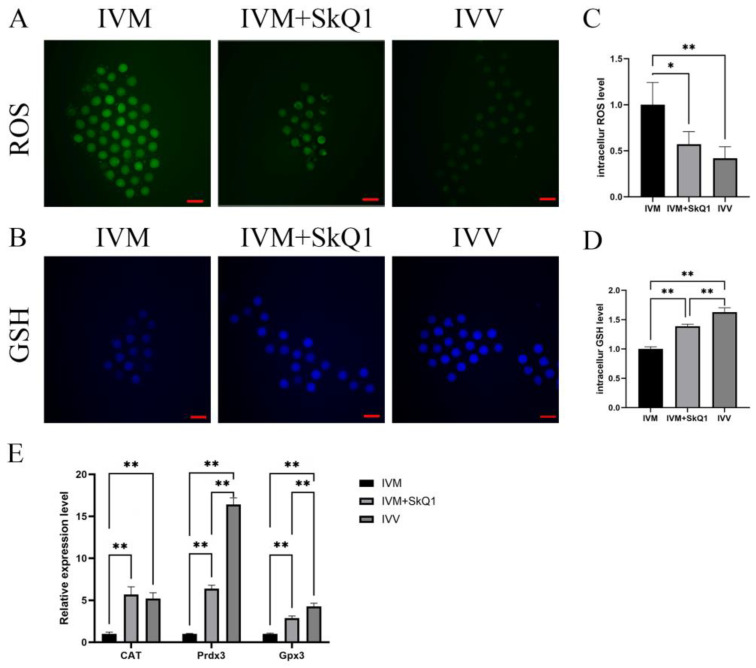
Antioxidant effects of SkQ1on in vitro-matured mouse oocytes. (**A**) Representative images of intracellular ROS (stained with DCFH-DA as green fluorescence) of three groups of oocytes. (**B**) Representative images of intracellular GSH (stained with CMF2HC as blue fluorescence) of three groups of oocytes. (**C**) Quantification data of fluorescence images representing ROS levels. (**D**) Quantification data of fluorescence images representing GSH levels. (**E**) qPCR analysis of three antioxidant genes, CAT, Prdx3 and Gpx3. * represents *p* < 0.05; ** represents *p* < 0.01. Scale bar: 50 μm. IVM—in vitro-matured oocytes; IVM + SkQ1—SkQ1-treated in vitro-matured oocytes; IVV—in vivo-matured oocytes.

To investigate the impacts of SkQ1 on alleviating oxidative stress in mouse oocytes during IVM, we assessed ROS and GSH levels, along with the mRNA expression levels of antioxidant genes GPx3, CAT, and Prdx3 in the IVM, IVM + SkQ1, and IVV groups of oocytes. The ROS level in the IVM + SkQ1 group of oocytes, which was similar to that in the IVV oocyte group, was significantly lower (*p* < 0.05) than that in the IVM group of oocytes (Figure 2A,C); The GSH level in the IVM + SkQ1 oocytes was significantly higher than that in the IVM oocytes, and was comparable to that in the IVV oocytes (Figure 2B,D). The transcription levels of three antioxidant genes, GPx3, CAT, and Prdx3,in the IVM + SkQ1 and IVV groups were significantly higher than those in the IVM oocytes (Figure 2E). These data imply that SkQ1 treatment during IVM significantly reduces oxidative stress levels and increases antioxidant capacity in mouse oocytes.

### 2.3. SkQ1 Increased Mitochondrial Membrane Potential in In Vitro-Matured Mouse Oocytes

**Figure 3:**
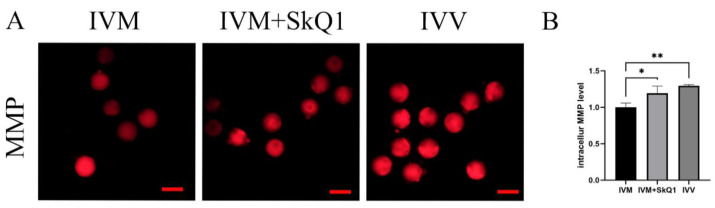
Effects of SkQ1 on mitochondrial membrane potential in in vitro-matured mouse oocytes. (**A**) Representative images of intracellular MMP (stained with TMRE as red fluorescence) of three groups of oocytes. (**B**) Quantification data of fluorescence images representing MMP levels. * represents *p* < 0.05; ** represents *p* < 0.01. Scale bar: 50 μm. IVM—in vitro-matured oocytes; IVM + SkQ1—SkQ1-treated in vitro-matured oocytes; IVV—in vivo-matured oocytes.

Oocyte quality is mainly determined by mitochondrial function [19,20], which can be evaluated by mitochondrial membrane potential [11]. Therefore, we examined the impacts of SkQ1 on the mitochondrial membrane potential during mouse in vitro oocyte maturation. The results indicated that the IVM + SkQ1 and IVV groups of oocytes had significantly higher mitochondrial membrane potential than the IVM oocytes (Figure 3). This result suggests that the addition of SkQ1 to mouse IVM medium can prevent mitochondrial dysfunction by increasing the mitochondrial membrane potential.

### 2.4. SkQ1 Inhibited Apoptosis in In Vitro-Matured Mouse Oocytes

**Figure 4:**
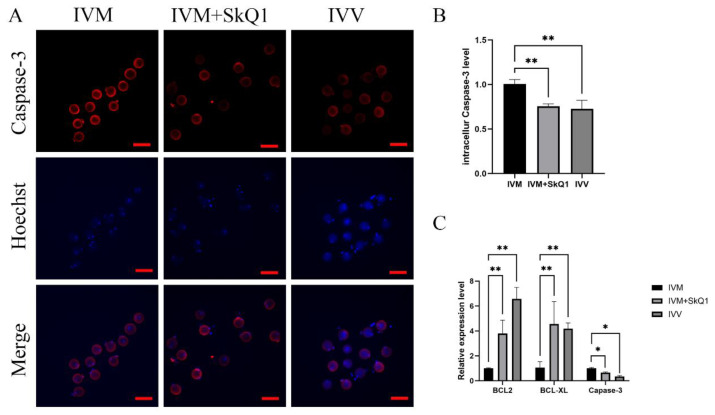
Effects of SkQ1 on apoptosis of in vitro-matured mouse oocytes. (**A**) Representative images of intracellular Caspase-3 protein of three groups of oocytes. (**B**) Quantification data of fluorescence images representing the Caspase-3 protein levels. (**C**) qPCR analysis of three antioxidant genes, BCL2, BCL-XL, and Caspase-3. * represents *p* < 0.05; ** represents *p* < 0.01. Scale bar: 50 μm. IVM—in vitro-matured oocytes; IVM + SkQ1—SkQ1-treated in vitro-matured oocytes; IVV—in vivo-matured oocytes.

To further investigate the impacts of SkQ1 on oocyte apoptosis during IVM, we evaluated the expression levels of several genes related to apoptosis. The results demonstrated that the addition of 0.01 μM SkQ1 to IVM medium significantly down-regulated (*p* < 0.05) the protein and mRNA levels of the pro-apoptotic gene Caspase-3 (Figure 4A–C) and markedly up-regulated (*p* < 0.01) the mRNA expression levels of the anti-apoptotic genes Bcl-2 and Bcl-XL (Figure 4C). Thus, it is evident that the supplementation of SkQ1 during mouse in vitro oocyte maturation can inhibit oocyte apoptosis.

## 3. Discussion

In the first experiment, we determined the optimal treatment concentration of SkQ1 by assessing the maturation rates of mouse oocytes cultured with different concentrations of SkQ1. We observed a significant increase in the oocyte maturation rate with the addition of 0.01 μM SkQ1 to the IVM medium. However, the supplementation of either 0.02 or 0.04 μM SkQ1 did not significantly affect the maturation rates of the treated oocyte groups. This finding supports the notion that maintaining a balance between pro-oxidants and antioxidants is crucial for oocyte development, as high concentrations of antioxidants can disrupt meiotic progression and result in reduced oocyte maturation rates [13,21]. Although our data showed that the addition of a low concentration of SkQ1 (0.01 μM) to the IVM medium effectively enhanced mouse oocyte maturation, whether treatment with a lower dose of SkQ1 (lower than 0.01 μM) would result in a greater enhancement in the oocyte maturation rate needs to be investigated in future studies.

During IVM, oocytes experience a reduction in antioxidant capacity, compared to that in the in vivo environment, rendering them more susceptible to oxidative stress, and resulting in elevated levels of ROS. This oxidative stress can lead to cell membrane damage, DNA breaks, and mitochondrial dysfunction, contributing to oocyte meiosis arrest and subsequent embryonic developmental disruption [22]. GSH is a non-enzymatic intrinsic antioxidant that is highly susceptible to oxidation due to its sulfhydryl group. It scavenges free radicals and protects the sulfhydryl groups of biological macromolecules, such as proteins and enzymes [10,22]. The level of GSH is crucial for the maturation of the oocyte cytoplasm, affecting the reducing capacity of the oocyte cytoplasm and inducing the formation of the male prokaryotic nucleus after fertilization [9,11]. This process plays an essential role in regulating an oocyte’s developmental capacity [23]. Previous research has indicated that an increase in the ROS level is often accompanied by a decrease in the GSH level, leading to the inhibition of in vitro oocyte maturation and resulting in fertilization failure [2]. Therefore, GSH is a key indicator for assessing the cytoplasmic maturation of oocytes [24]. Our results revealed that the addition of 0.01 μM SkQ1 significantly increased oocyte GSH levels, approaching those of in vivo-matured oocytes. This aligns with the effects observed with another mitochondria-targeted antioxidant, MitoQ [2,11].

The functional state of the mitochondria is critical for providing sufficient energy to sustain mitotic spindle assembly and release the polar body in oocytes [25]. Furthermore, embryonic development following fertilization is a highly energy-intensive process, primarily relying on mitochondrial oxidative phosphorylation to produce ATP. As a result, the integrity of the mitochondrial membrane and its capacity to generate adequate ATP play vital roles in determining the maturation of the oocyte and the development of the early embryo. SkQ1 is a unique mitochondrial antioxidant. It can enter the mitochondria to improve the redox signaling pathway, preventing mitochondrial damage and peroxidation of the mitochondrial membrane lipids [16,17]. This ensures sufficient energy generation during oocyte maturation, leading to an increase in the maturation rates of oocytes and an improvement in the developmental efficiency of the subsequent embryos, as observed in the oocytes treated with antioxidant SkQ1 in the present study. Our results are consistent with those of previous studies, suggesting that the addition of antioxidants such as melatonin [12], vitamin E, vitamin C [14], quercetin [15], and L-carnitine [26] can promote in vitro oocyte maturation and subsequent embryonic development by modulating the mitochondrial membrane potential [10,11].

High levels of ROS generated by oxidative stress contribute to the occurrence of apoptosis by impairing mitochondrial activity in oocytes and embryos. Treatment with SkQ1 increased the mRNA abundance of anti-apoptosis genes Bcl-2 and Bcl-xl, and de-creased the mRNA and protein levels of pro-apoptosis gene Caspase-3 in mouse oocytes. Galkin’s initials also discovered that adding SkQ1 to the culture medium of mouse endothelial cells increased the expression levels of Bcl-2 and Bcl-xl, while decreasing the expression of Caspase-3 [17,27]. These results are consistent with the findings of the present study, which indicate that SkQ1 has an inhibitory effect on apoptosis.

## 4. Materials and Methods

### 4.1. Ethics Statement

In this study, 6–8-week-old SPF ICR mice (supplier: Southern Medical University Experimental Animal Center, Guangzhou, China) were utilized. The animal study protocol was approved by the Ethics Committee of the Experimental Animal Center of South China Agricultural University (License No: SYXK-2019-0136).

### 4.2. Germinal Vesicle Oocyte Collection

Female ICR mice were injected intraperitoneally, at 6–8 weeks old, with 5–10 IU of PMSG (Ningbo No. 2 Hormone Factory, Ningbo, China). After 48 h, their ovaries were removed and placed in pre-warmed M2 culture medium (MR-015, Sigma, St. Louis, MO, USA) at 37 °C. Under aseptic conditions, excess adipose tissue was removed from each ovary, and the ovary was repeatedly punctured with a 1 mL syringe needle until it became a paste, without large pieces of tissue. Germinal vesicle (GV) oocytes enclosed within the intact compact cumulus layers were selected and transferred to IVM culture medium (M2115, Aibei, Nanjing, China).

### 4.3. Oocyte In Vitro Maturation and Drug Treatment

GV-stage oocytes were collected and cultured in IVM culture medium at 37 °C in an incubator containing 5% CO_2_ for 18–20 h. the experimental groups were supplemented with 0.01, 0.02, or 0.04 µM SkQ1, while the in vitro control group was supplemented with the same volume of dimethylsulfoxide (as a solvent for SkQ1).

After incubation with SkQ1 or dimethylsulfoxide for 18–20 h, oocytes with one polar body (PB) were verified as metaphase II (MII oocytes). The number of MII oocytes with a round zona pellucida (ZP), a small perivitelline space, and a pale moderately granular cytoplasm without inclusions was counted for each group [28]. The optimal concentration of SkQ1 for treatment was determined, and subsequent experiments were validated using this concentration.

### 4.4. Collection of In Vivo-Matured Oocytes

The 6–8-week-old female ICR mice were injected intraperitoneally with 5–10 IU PMSG, followed by 5–10 IU hCG 48 h later. The oviducts were removed 16–18 h after hCG injection under aseptic conditions. The ampulla segment of each oviduct was cut open using a 1 mL syringe needle to collect M II oocytes from the bottom of the dish [29].

### 4.5. Experimental Design

To investigate the impacts of different concentrations of SKQ1 on the maturation rates of in vitro-matured mouse oocytes, oocytes were divided into 4 groups, including 3 SKQ1 treatment groups, which were respectively supplemented with 0.01, 0.02, or 0.04 µM SkQ1 during IVM, and one control group (IVM group) that was supplemented with the same volume of dimethylsulfoxide (the solvent for SkQ1).

To investigate the effects of the addition of SKQ1 during mouse IVM on subsequent IVF embryonic development, the oocytes were divided into three groups, including one negative control group that was not treated with SKQ1 during IVM (IVM group), one SKQ1 treatment group that was supplemented with 0.01 µM SKQ1 during IVM (IVM + SKQ1 group), and one positive control group that was derived from in vivo-matured oocytes (IVV group). The same three groups of oocytes were used in the experiments for studying the effects of addition of SKQ1 during mouse IVM on mitochondrial membrane potential, intracellular GSH levels, ROS production, and apoptosis in treated mouse oocytes.

### 4.6. In Vitro Fertilization

HTF medium (M1135, Aibei, Nanjing, China) and TYH medium (M2035, Aibei, Nanjing, China), covered with mineral oil, were pre-cultured at 37 °C and 5% CO_2_ for 4 h in humid air. Healthy ICR male mice were then selected, and their epididymal tail and vas deferens were removed. The removed tissues were washed with PBS to remove fat and blood, and then manipulated under a stereomicroscope to obtain spermatozoa, which were placed into the pre-equilibrated TYH, and incubated for 50 min (37 °C, 5% CO_2_). The sperm concentration was adjusted to 4 × 10^5^ sperm cells/mL, and then transferred into HTF, where the oocytes had been placed. After 5–6 h, oocytes with two pronuclei were washed with DPBS, moved to KSOM medium (M1435, Aibei, Nanjing, China), and cultured for further development (37 °C, 5% CO_2_). The cleavage rates and blastocyst rates were counted at 24 h and 120 h post fertilization, respectively [30].

### 4.7. Quantitative PCR

Twenty oocytes that were mixed into one sample were lysed in 100 μL RTL Lysis Buffer (containing 14.3 M β-mercaptoethanol and 1 μg/μL Carrier RNA). RNA was then extracted using the HiPure Total RNA Nano Kit (R4125-02, Magen, Guangzhou, China), and cDNA was synthesized using the Evo M-MLV RT Mix Kit with gDNA Clean for qPCR (AG11728, Accurate Biology, Changsha, China). qPCR was performed using the SYBR Green Premix Pro Taq HS qPCR Kit (AG11718, Accurate Biology, Changsha, China).

Based on the gene sequences of GPx3, CAT, Prdx3, Bcl2, Bcl-XL, and Caspase-3 from the National Center for Biotechnology Information, primers were designed using Primer 3 Plus software, and the primer information is shown in Table 1. The primers were all synthesized by the BGI-Huada Genomics company (Shenzhen, China).

The qPCR reaction system was 20 μL, comprised as follows: cDNA 1 μL, SYBR Mix 10 μL, each upstream and downstream primer 0.8 μL, ROX Low 0.4 μL, and ddH2O 7.8 μL. The qPCR reaction procedure was as follows: 95 °C for 3 s, 60 °C for 20 s, and 72 °C for 1 s. The relative expression of each gene was calculated using the 2^−ΔΔCt^ method [31].

### 4.8. ROS Level Measurement

The ROS levels in oocytes were evaluated using an ROS assay kit (S0033S, Beyotime, Shanghai, China). The oocytes were placed in DCFH-DA, diluted with M2 medium (1:1000), and incubated at 37 °C and 5% CO_2_ for 30 min, protected from light, and then washed with DPBS 3 times. The samples were observed under a Nikon eclipse Ti-S microscope (ti-2U, Nikon, Tokyo, Japan).

### 4.9. GSH Level Measurement

The GSH levels in oocytes were evaluated using CMF2HC (C12881, Invitrogen, Carlsbad, CA, USA). The oocytes were placed in CMF2HC, diluted with M2 medium (1:1000), and incubated at 37 °C and 5% CO_2_ for 30 min, protected from light, and then washed with DPBS 3 times. The samples were observed under a Nikon eclipse Ti-S microscope (ti-2U, Nikon, Tokyo, Japan).

### 4.10. Mitochondrial Membrane Potential Measurement

The mitochondrial membrane potential was measured using a Mitochondrial Membrane Potential Assay Kit with TMRE (C2001S, Beyotime, Shanghai, China). The oocytes were placed in TMRE, diluted with M2 medium (1:1000), and incubated at 37 °C and 5% CO_2_ for 30 min, protected from light, and then washed with DPBS 3 times. The samples were observed under a Nikon eclipse Ti-S microscope (ti-2U, Nikon, Tokyo, Japan).

### 4.11. Immunofluorescence

Twenty oocytes in each group were fixed with Immunol Staining Fix Solution (P0098, Beyotime, Shanghai, China) for 15 min at room temperature. They were then permeabilized with 1% TritonX-100 (P0096, Beyotime, Shanghai, China) for 20 min and incubated with immunofluorescence-blocking solution (P0228, Beyotime, Shanghai, China) for 30 min. The oocytes were subsequently incubated with Caspase-3 (A0214, Abclonal, Wuhan, China) antibody, diluted with QuickBlock™ Immunostaining Primary Antibody Dilution Solution (P0262, Beyotime, Shanghai, China), at 1:100, for 12 h at 4 °C. The oocytes were incubated with goat anti-rabbit IgG(H + L) (A0214, Beyotime, Shanghai, China), diluted with Immunol Fluorescence Staining Secondary Antibody Dilution Buffer (P0108, Beyotime, Shanghai, China), at a 1:200 ratio for 1 h at room temperature. After incubation, the oocytes were stained with Hochest 33342 for 10 min, and then washed with DPBS 3 times. The samples were observed under a Nikon eclipse Ti-S microscope (ti-2U, Nikon, Tokyo, Japan).

### 4.12. Statistical Analysis

Each experiment in this study was repeated at least three times. All data were statistically analyzed using Graphpad Prism 10.1.2 with one-way ANOVA or *t*-test. The experimental data are expressed as mean ± SEM. A statistically significant result was considered when *p* < 0.05.

## 5. Conclusions

The addition of antioxidant SkQ1 to the IVM medium improves the mouse oocyte maturation rate and the subsequent IVF embryonic developmental capacity. SkQ1 treatment also reduces oxidative stress and apoptosis, and increases the mitochondrial membrane potential of in vitro-matured oocytes. This study establishes a new method through which to improve the maturation rates and quality of in vitro-matured mouse oocytes, which could benefit the application and development of assisted reproductive technology in both animals and humans.

## Figures and Tables

**Table 1:** Primer Information.

Genes	Primer Sequences (5′ → 3′)	Product Length (bp)	GenBank Acc No.	Annealing Temp (°C)
**Gapdh**	F:CAGTCCATGCCATCACTGCCA R:ATGCCAGTGAGCTTCCCGTTC	163	NC_000072	59.76
**CAT**	F:GACAAAATGCTTCAGGGCCGC R:ACCCTGGTTGTCATGCATGCA	156	NC_009804	58.00
**Prdx3**	F:GTGCTGTTGGAAAGTGCTGGC R:TGGGCAGACTTCTCCATGGGT	171	NM_007452	59.76
**Gpx3**	F:AAACAGGAGCCAGGCGAGAAC R:AGTGGGAGGGCAGGAGTTCTT	162	NM_001329860	59.76
**Bcl2**	F:GCATCTGCACACCTGGATCCA R:ACTTGTGGCCCAGGTATGCAC	170	NM_009741	59.50
**Bcl-XL**	F:TTTTTCTCCTTTGGCGGGGCA R:TCCACAAAAGTGTCCCAGCCG	152	NM_001289716	59.50
**Caspase-3**	F:TTCATCATTCAGGCCTGCCGG R:TGAACCACGACCCGTCCTTTG	175	NM_001284409	59.76

## Data Availability

The data that support the findings of this study are available on request from the corresponding author (Z.L.), upon reasonable request.

## Abbreviations

IVM	In vitro oocyte maturation
ETC	Electron transport chain
DNA	Deoxyribonucleic acid
RNA	Ribonucleic acid
mtDNA	Mitochondrial DNA
ICR	Institute of Cancer Research
PMSG	Pregnant mare serum gonadotropin
hCG	Human chorionic gonadotropin
PB	Polar body
ZP	Zona pellucida
IVF	In vitro fertilization
qPCR	Quantitative real-time PCR
MMP	Mitochondrial membrane potential
GSH	Glutathione
DPBS	Dulbecco’s Phosphate-Buffered Saline
COCs	Cumulus–oocyte complexes
GV	Germinal vesicle
MII	Metaphase II
DMSO	Dimethyl sulfoxide
HTF	Human tubal fluid
h	Hour
m	Minute
ROS	Reactive oxygen species
TMRE	Tetramethylrhodamine
%	Percentage
